# Supplementary material for: Systematic identification of bacterial factors driving Staphylococcus aureus intracellular lifestyle in non-professional phagocytes
Source: Nat Commun. 2025 Dec 10;16:10907. doi: 10.1038/s41467-025-66373-9 (PMC12696020; doi:10.1038/s41467-025-66373-9)
Supplement: Supplementary file 1 — Supplementary Information [file 41467_2025_66373_MOESM1_ESM.pdf]

SUPPLEMENTARY FIGURES

Systematic identification of bacterial factors driving *Staphylococcus aureus* intracellular lifestyle in non-professional phagocytes

Ines Rodrigues Lopes, Laura Maria Alcantara, Maria Lopez-Bravo, Yi Liu,
Gerald Larrouy-Maumus, Daniel Lopez, Miguel Mano, Ana Eulalio

*Corresponding authors
e-mail: miguel.mano@kcl.ac.uk ; ORCID: 0000-0003-1922-4824
e-mail: a.eulalio@imperial.ac.uk ; ORCID: 0000-0002-7355-0674

Rodrigues Lopes *et al.* - Supplementary Figure 1

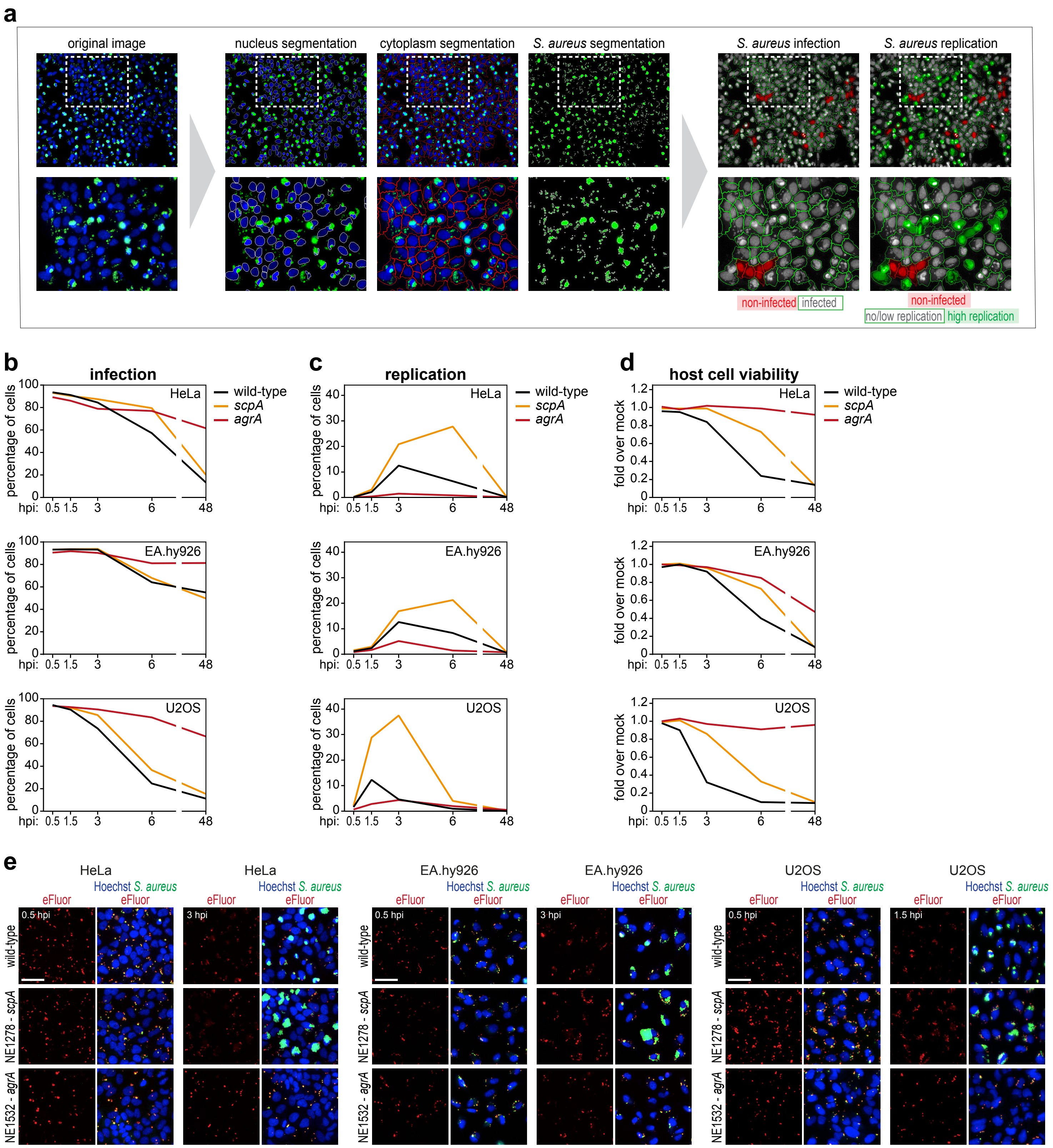

Supplementary Figure 1. Optimization of the microscopy-based infection assay used for the *S. aureus* NTML high-throughput screening.

a. Fluorescence microscopy image, corresponding image segmentation outlines, and classification (infection, replication) of HeLa cells infected with *S. aureus* WT (USA300 JE2). In the infection classification panel, cells shaded in red and outlined in green correspond to non-infected and infected cells, respectively; in the replication classification panel, cells shaded in red correspond to non-infected cells, cells outlined in green correspond to cells with no/low *S. aureus* replication and cells shaded in green correspond to cells showing high S. aureus replication.

b-d. Time-course analysis of infection (**b**), intracellular replication (**c**), and host cell viability (**d**) upon infection of HeLa, EA.hy926 and U2OS cells with *S. aureus* WT and the mutant strains *agrA* and *scpA*, used for optimization and as controls in all assay plates. For each strain, results are presented as the mean of 3 biologically independent experiments.

e. Representative images of eFluor proliferation assay, upon infection of HeLa, EA.hy926, and U2OS cells with *S. aureus* WT and the mutant strains *agrA* and *scpA*. Infection was analysed at 0.5 hpi (all cell types), 1.5 hpi (U2OS) and 3 hpi (HeLa and EA.hy926 cells). Microscopy images are representative of 3 biologically independent experiments. Scale bar, 50 µm.

Source data are provided as a Source Data file.

Rodrigues Lopes *et al.* - Supplementary Figure 2

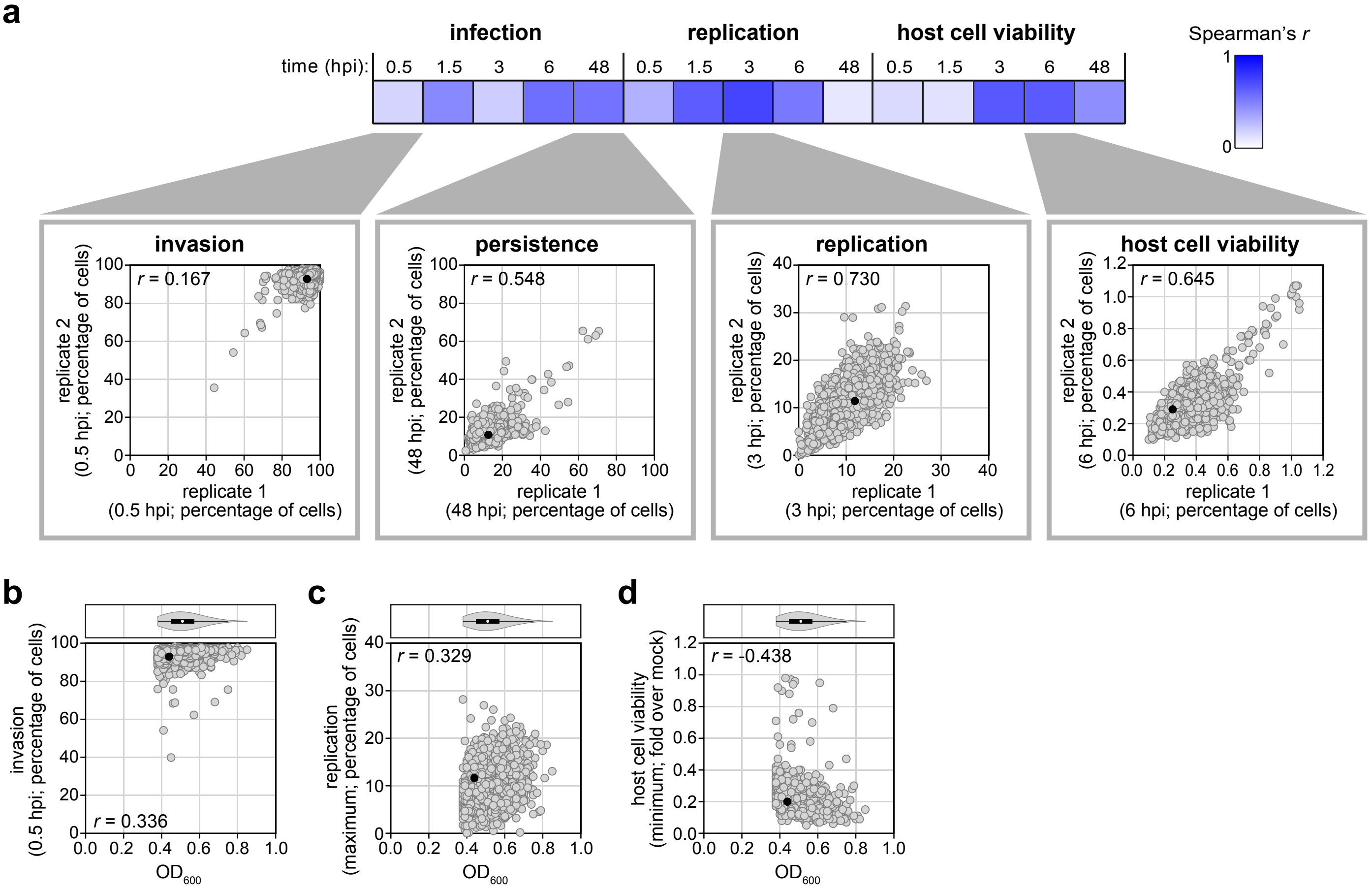

Supplementary Figure 2. Readouts of the *S. aureus* NTML screening show high reproducibility between the two independent experiments.
a. Heat map showing Spearman's rank correlation coefficients and pairwise comparison between the two independent runs of the screening (1,920 mutant strains). For the heat map, results are shown for all time points tested, and for the pairwise correlations results are shown for selected endpoints: invasion (infection 0.5 hpi), persistence (infection 48 hpi), bacterial intracellular replication (3 hpi), and host cell viability (6 hpi). Cells infected with *S. aureus* WT are shown for comparison (black dot). Spearman's rank correlation coefficients are shown in the upper left corner of each graph.
b-d. Comparison of OD_{600} of the NTML bacterial growths used for infection and the percentage of host cells with *S. aureus* invasion (infection at 0.5 hpi; **b**), intracellular replication (maximum; **c**), or host cell viability (minimum; **d**). Cells infected with *S. aureus* WT are shown for comparison (black dot). Results shown for each strain correspond to the mean of the 2 biologically independent experiments performed. The uppermost plots (violin plots) show the overall distribution of OD_{600} across the NTML; white circles show the medians, box limits indicate the 25th-75th percentiles, whiskers extend 1.5 times the interquartile range from the 25th and 75th percentiles, polygons extend to extreme values. Spearman's rank correlation coefficients are shown in the upper/bottom left corner of each graph.

Rodrigues Lopes *et al.* - Supplementary Figure 3

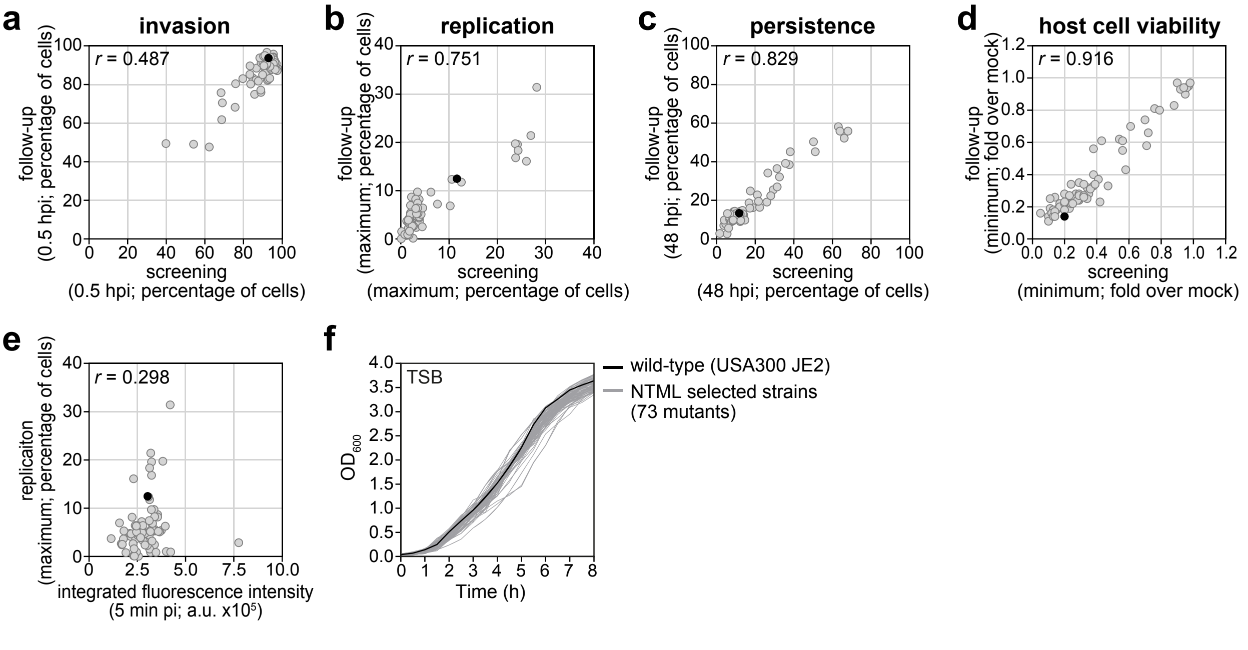

Supplementary Figure 3. Variations in *S. aureus* growth or host invasion do not account for the differences in replication observed for the 73 selected mutant strains.

a-d. Pairwise comparison of the results of the screening and follow-up experiments upon infection of HeLa cells with the 73 selected *S. aureus* mutants, for invasion (infection at 0.5 hpi, **a**), intracellular replication (maximum; **b**), persistence (infection at 48 hpi; **c**) and host cell viability (minimum; **d**). Cells infected with *S. aureus* WT are shown for comparison (black dot). Results are shown as the mean of 2 biologically independent experiments for the screening, and the mean of 3 biologically independent experiments for the follow-up experiments. Spearman's rank correlation coefficients are shown in the upper left corner of each graph.

e. Comparison of the integrated intensity of bacterial fluorescence signal (proxy for bacterial intracellular load) at 5 min pi and the maximum of intracellular replication upon infection of HeLa cells with the 73 selected *S. aureus* strains. Cells infected with *S. aureus* WT are shown for comparison (black dot). Spearman's rank correlation coefficient is shown in the upper left corner of the graph.

f. Growth curves of the 73 selected *S. aureus* mutants in TSB. *S. aureus* WT is shown for comparison (black line). OD_{600} was measured at 30 min intervals for 8 h. For each strain, results are presented as the mean of 3 biologically independent experiments.

Source data are provided as a Source Data file.

Rodrigues Lopes *et al.* - Supplementary Figure 4

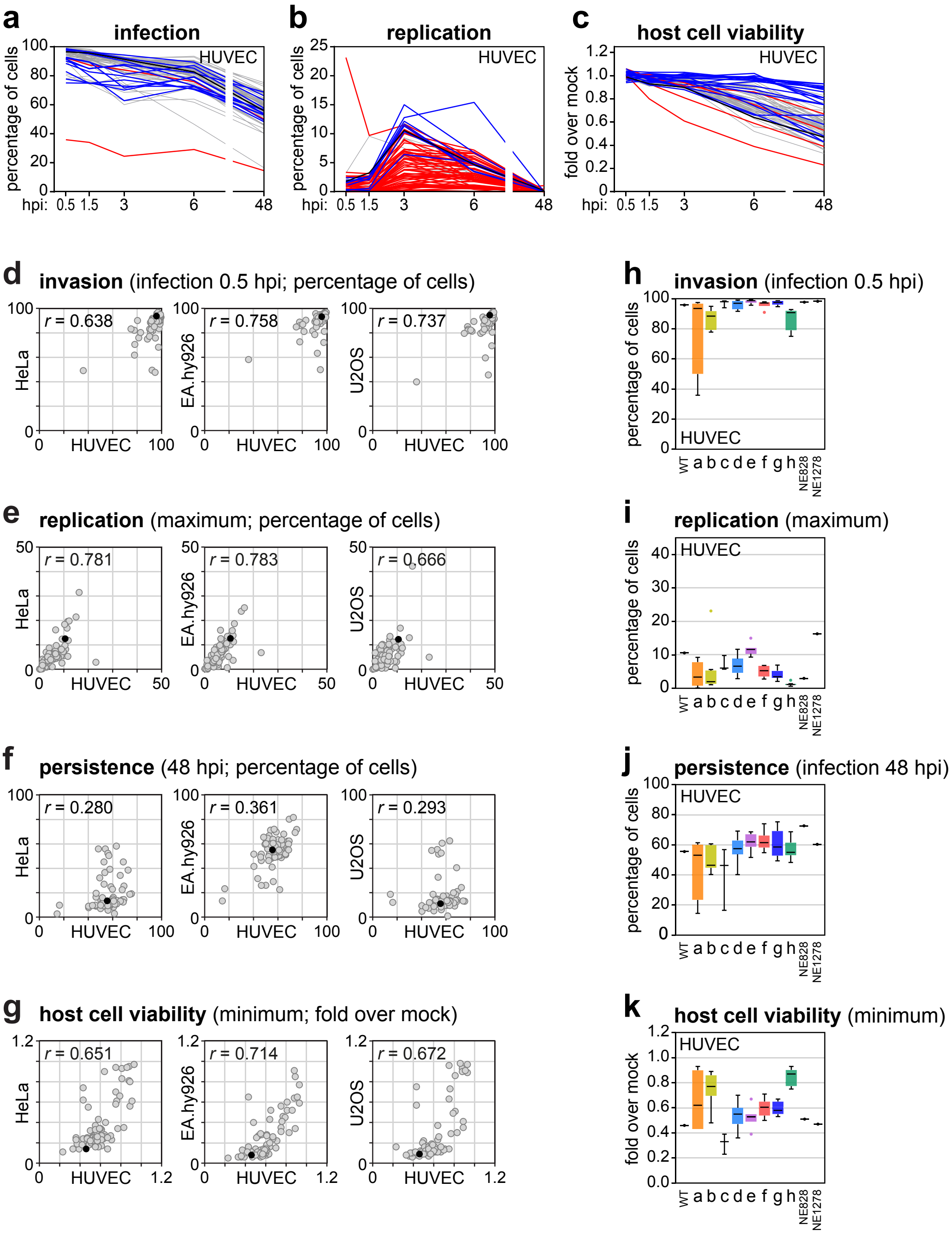

Supplementary Figure 4. *S. aureus* intracellular infection phenotypes were validated upon infection of primary cells.

a-c. Time-course analysis of infection (**a**), intracellular replication (**b**), and host cell viability (**c**) for the 73 selected *S. aureus* mutants upon infection of primary endothelial cells (HUVEC). Strains highlighted in blue and red correspond to the strains selected from the screening, highlighted in Fig.1b-d. Cells infected with *S. aureus* WT are shown for comparison (black line). For each strain, results are presented as the mean of 3 biologically independent experiments.

d-g. Pairwise comparison of the percentage of invasion (infection at 0.5 hpi; **d**), intracellular replication (maximum; **e**), persistence (infection at 48 hpi; **f**), and host cell viability (minimum; **g**) upon infection of the HeLa, EA.hy926, U2OS and HUVEC cells. Cells infected with *S. aureus* WT are shown for comparison (black dot). Results are presented as the mean of 3 biologically independent experiments. Spearman's rank correlation coefficients are shown in the upper left corner of each graph.

h-k. Box-plots showing the distribution of data for *S. aureus* WT and 73 selected mutant strains concerning invasion (infection at 0.5 hpi; **h**), intracellular replication (maximum value; **i**), persistence (infection at 48 hpi; **j**), and host cell viability (minimum; **k**) upon infection of HUVEC. Results are coloured by phenotypic group; box-plots were generated using the mean of 3 biologically independent experiments per strain; white lines show the medians, box limits indicate the 25th-75th percentiles, whiskers extend 1.5 times the interquartile range from the 25th and 75th percentiles.

Rodrigues Lopes *et al.* - Supplementary Figure 5

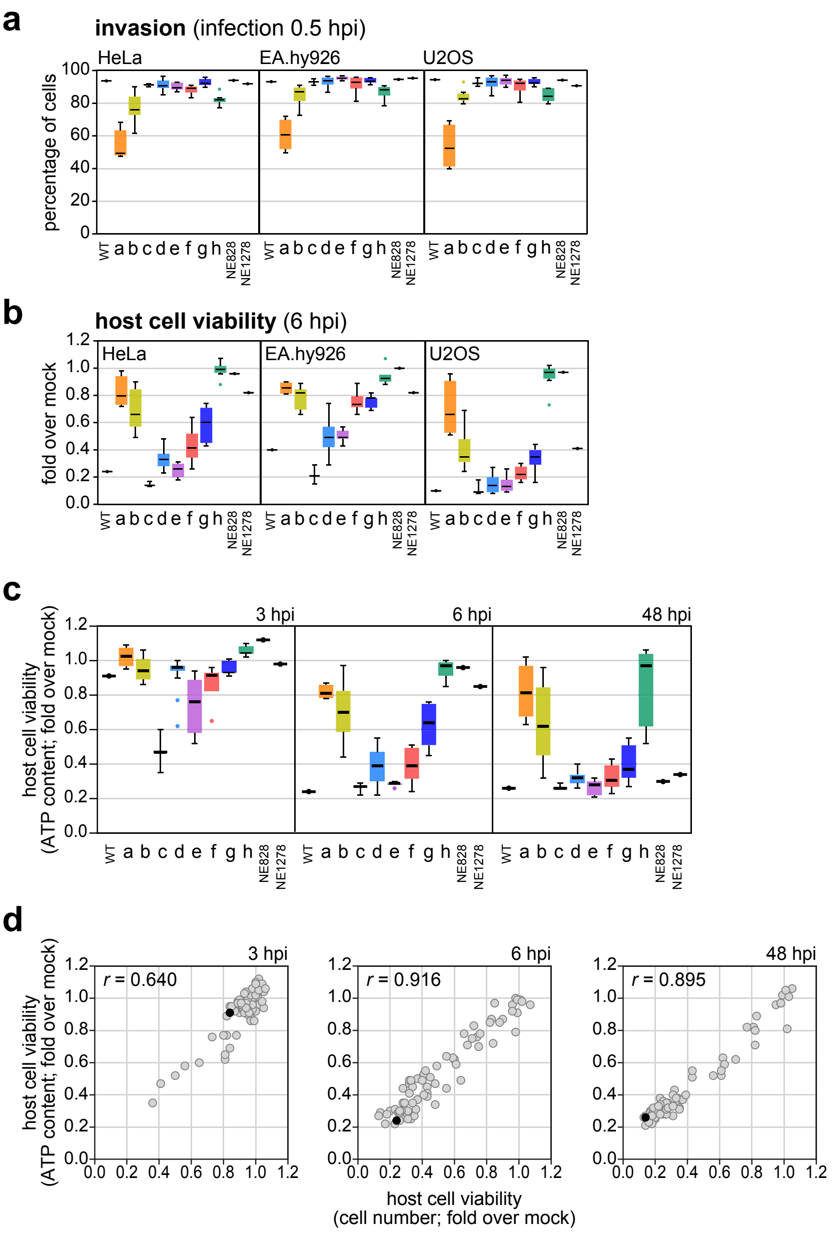

Supplementary Figure 5. Host cell viability phenotypes upon *S. aureus* infection were validated by ATP quantification.
a and b. Box-plots showing the distribution data for *S. aureus* WT and the 73 selected mutant strains concerning invasion (infection at 0.5 hpi; **a**), and host cell viability (6 hpi; **b**) upon infection of each of the three non-professional phagocytic cells. Box-plots were generated using the mean of 3 biologically independent experiments per strain, and are stratified by phenotypic group; white lines show the medians, box limits indicate the 25th-75th percentiles, whiskers extend 1.5 times the interquartile range from the 25th and 75th percentiles.
c. Box-plots showing the distribution of data for quantification of host cell viability determined by a luminescence-based ATP content assay, upon infection of HeLa cells with *S. aureus* WT and the 73 selected mutant strains. Box-plots were generated using the mean of 3 biologically independent experiments per strain, and are colored by phenotypic group; white lines show the medians, box limits indicate the 25th-75th percentiles, whiskers extend 1.5 times the interquartile range from the 25th and 75th percentiles.
d. Pairwise comparison of the percentage of host cell viability determined by microscopy-based infection assays and luminescence ATP content assays following infection of HeLa cells with *S. aureus* WT (black dot) and the 73 selected mutant strains, at three times post-infection (3, 6, and 48 hpi). Results are presented as the mean of 3 biologically independent experiments. Spearman's rank correlation coefficients are shown in the upper left corner of each graph.
Source data are provided as a Source Data file.

Rodrigues Lopes *et al.* - Supplementary Figure 6

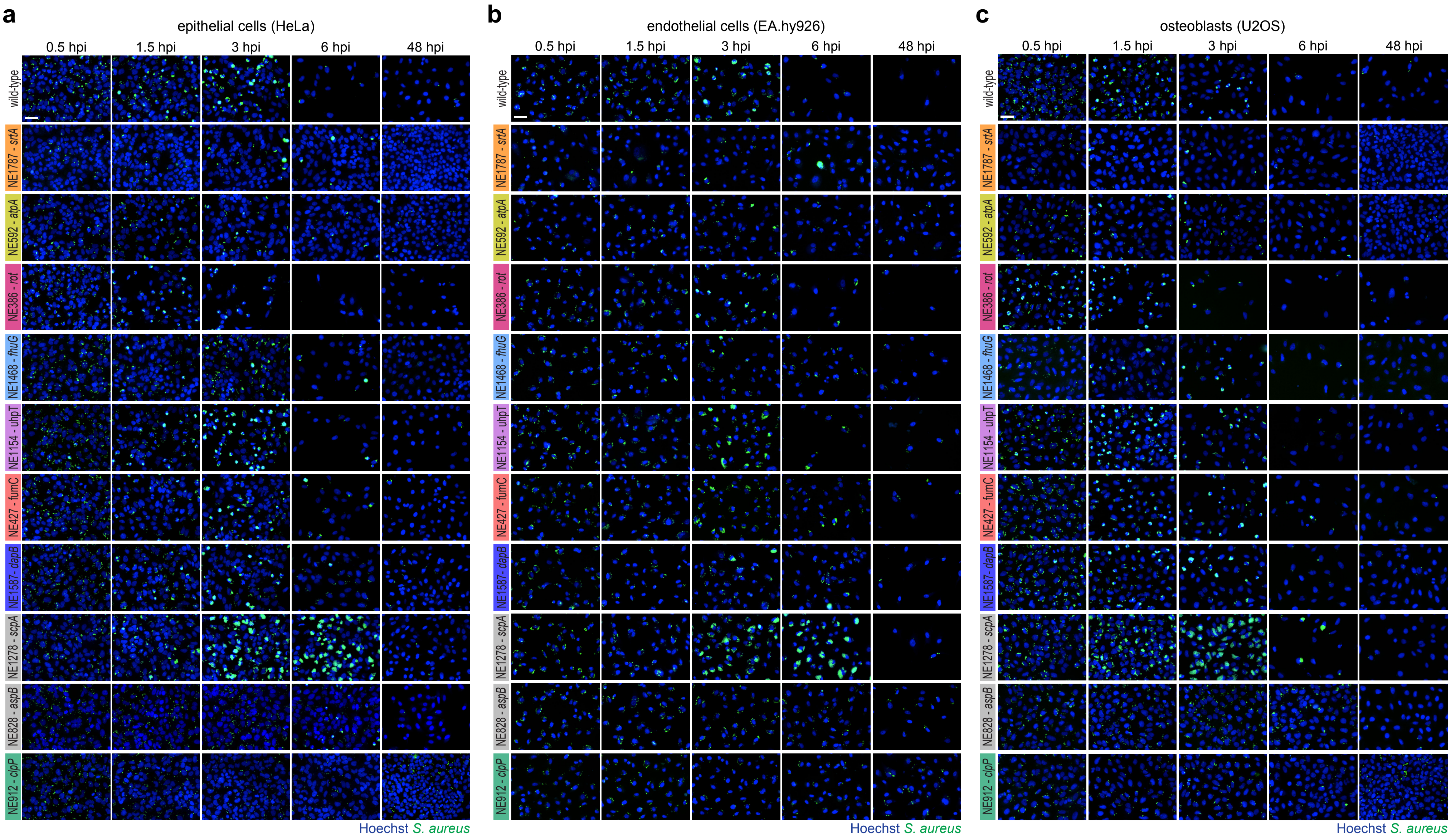

Supplementary Figure 6. Intracellular profiles of *S. aureus* strains belonging to distinct phenotypic clusters.
a-c. Representative fluorescence microscopy images of infection of epithelial cells (HeLa; **a**), endothelial cells (EA.hy926; **b**), and osteoblasts (U2OS; **c**) with selected S. aureus mutant strains belonging to the eight phenotypic profile clusters identified in Fig.3a, at 5 times post-infection (0.5, 1.5, 3, 6, and 48 hpi); images are also shown for infection with WT strain and with the two mutant strains not included in any of the clusters (strains NE828-*aspB* and NE1278-*scpA*). Images are representative of 3 biologically independent experiments. Scale bar, 50 µm.

Rodrigues Lopes *et al.* - Supplementary Figure 7

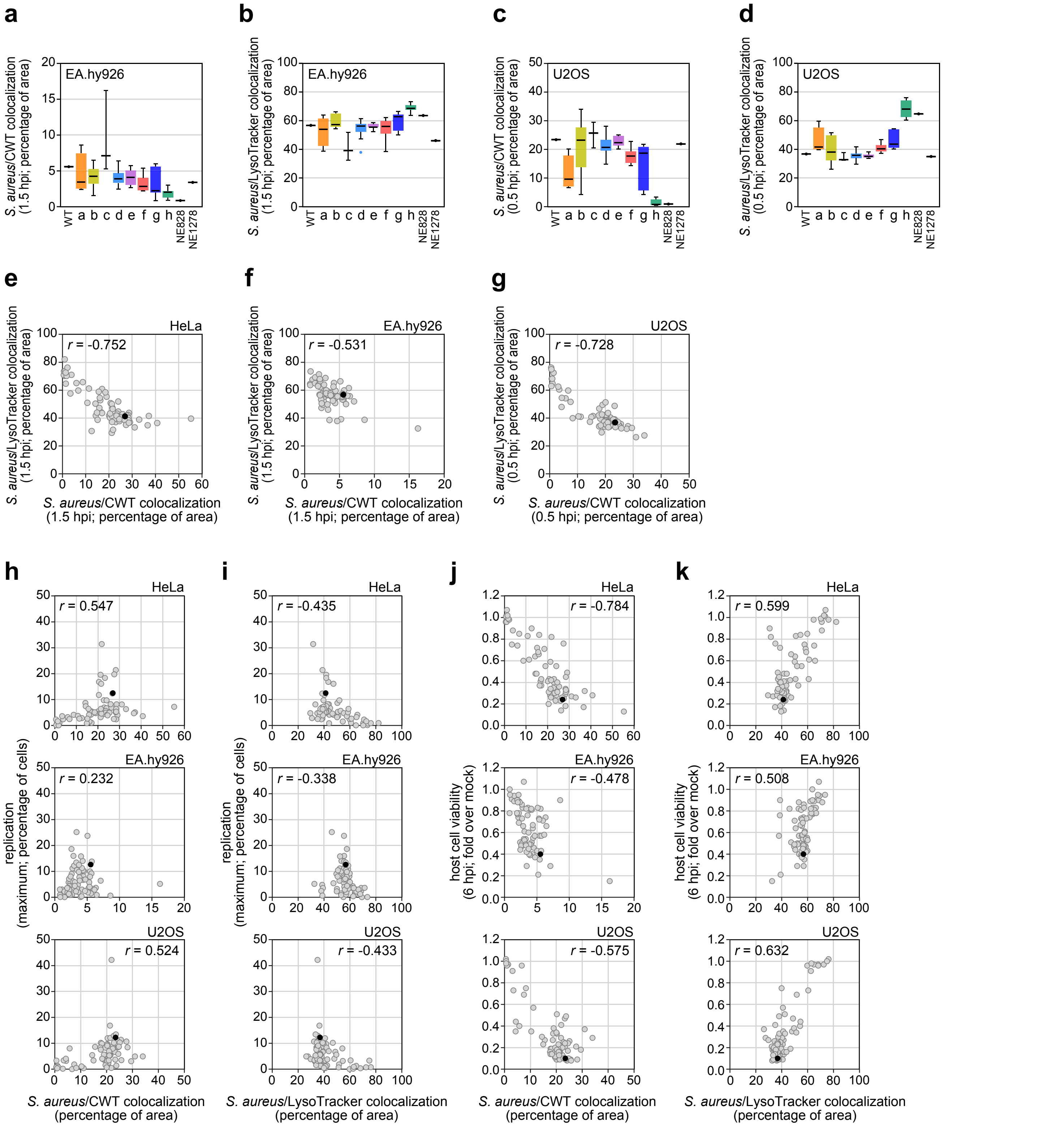

Supplementary Figure 7. *S. aureus* escape from the vacuole correlates with induction of host cell death.
a-d. Box-plots showing the distribution of data for *S. aureus* WT and 73 selected mutant strains concerning *S. aureus*/CWT (**a**,**c**) and *S. aureus*/LysoTracker (**b**,**d**) colocalization in EA.hy926 (**a**,**b**) and U2OS (**c**,**d**) cells analyzed at 1.5 hpi (EA.hy926) or 0.5 hpi (U2OS). Results are coloured by phenotypic group; box-plots were generated using the mean of 3 biologically independent experiments per strain; white lines show the medians, box limits indicate the 25th-75th percentiles, whiskers extend 1.5 times the interquartile range from the 25th and 75th percentiles.
e-g. Pairwise comparison of *S. aureus*/CWT and *S. aureus*/LysoTracker colocalizations upon infection of HeLa (1.5 hpi; **e**), EA.hy926 (1.5 hpi; **f**), and U2OS (0.5 hpi; **g**) with *S. aureus* WT (black dot) and the 73 selected mutant strains. Results are shown as the mean of 3 biologically independent experiments. Spearman's rank correlation coefficients are shown in the upper left corner of each graph.
h-k. Pairwise comparison of *S. aureus* maximum intracellular replication (**h**,**i**) and host cell viability (**j**,**k**) with *S. aureus*/CWT colocalization (**h**,**j**) or *S. aureus*/LysoTracker colocalization (**l**,**k**) upon infection of HeLa, EA.hy926 and U2OS cells with *S. aureus* WT (black dot) and the 73 selected mutant strains. Results are shown as the mean of 3 biologically independent experiments. Spearman's rank correlation coefficients are shown in the upper left/right corner of each graph.

Rodrigues Lopes *et al.* - Supplementary Figure 8

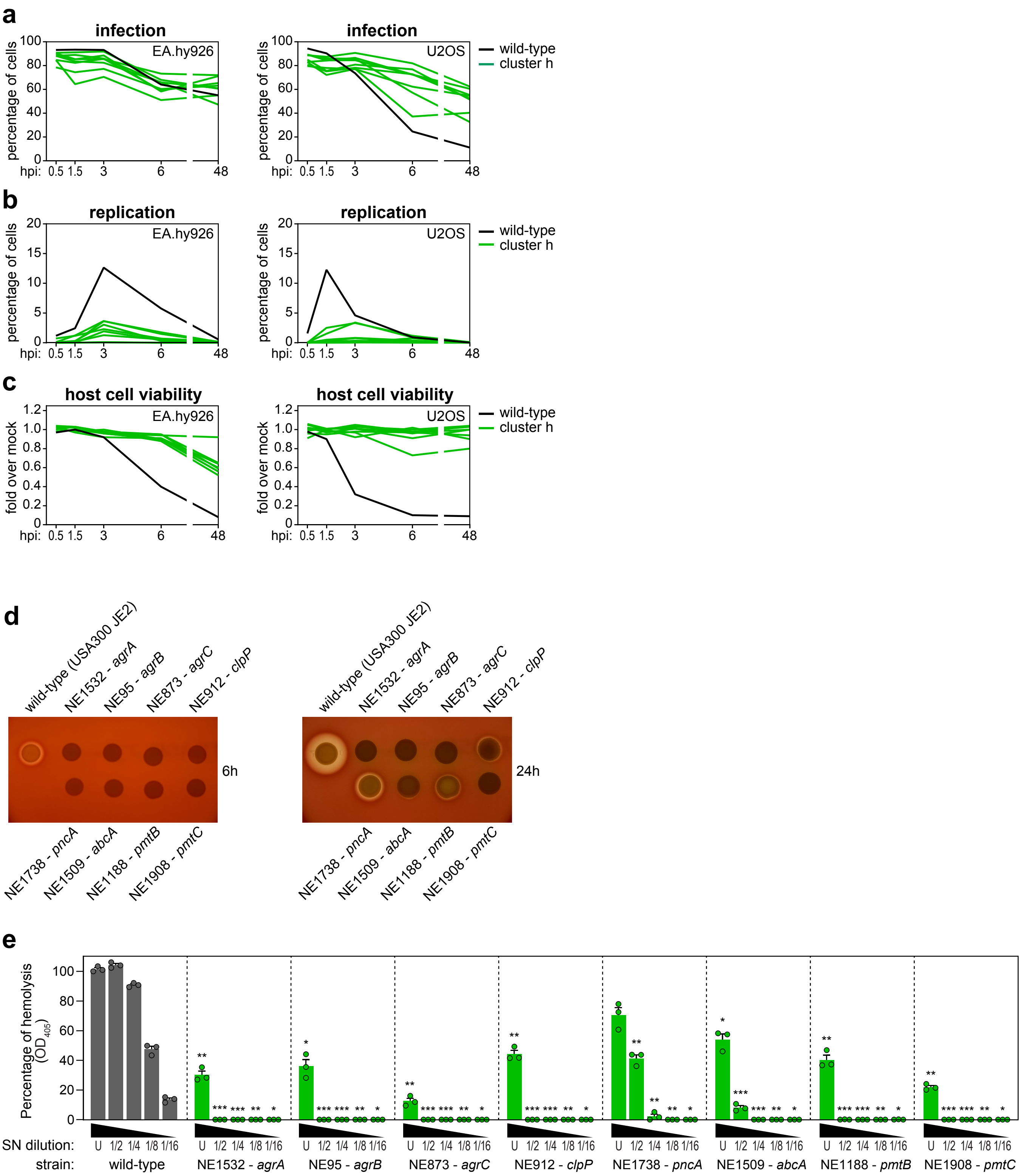

Supplementary Figure 8. *S. aureus* strains belonging to cluster h present low hemolytic activity.
a-c. Time-course analysis of infection (**a**), intracellular replication (**b**), and host cell viability (**c**) upon infection of EA.hy926 or U2OS cells with the 8 *S. aureus* mutant strains belonging to cluster h (green lines). Cells infected with *S. aureus* WT are shown for comparison (black line). For each strain, results are presented as the mean of 3 biologically independent experiments.
d and e. Hemolytic activity of *S. aureus* WT and the 8 mutant strains belonging to cluster h upon spotting of liquid cultures onto TSB agar containing 2% sheep blood (**d**) or quantification by measuring OD_{405} of a 2% sheep blood solution upon incubation with the strains supernatants (**e**). Culture supernatants were used undiluted (U) or after 2-fold serial dilutions (up to 1/16). All values are shown normalized to the OD_{405} of blood incubated with TSB containing 1% Triton X-100, and presented as mean ± s.e.m. of 3 biologically independent experiments; *P<0.05, **P<0.01, and ***P<0.001 (statistical analysis is detailed in Supplementary Data 4).
Source data are provided as a Source Data file.

Rodrigues Lopes *et al.* - Supplementary Figure 9

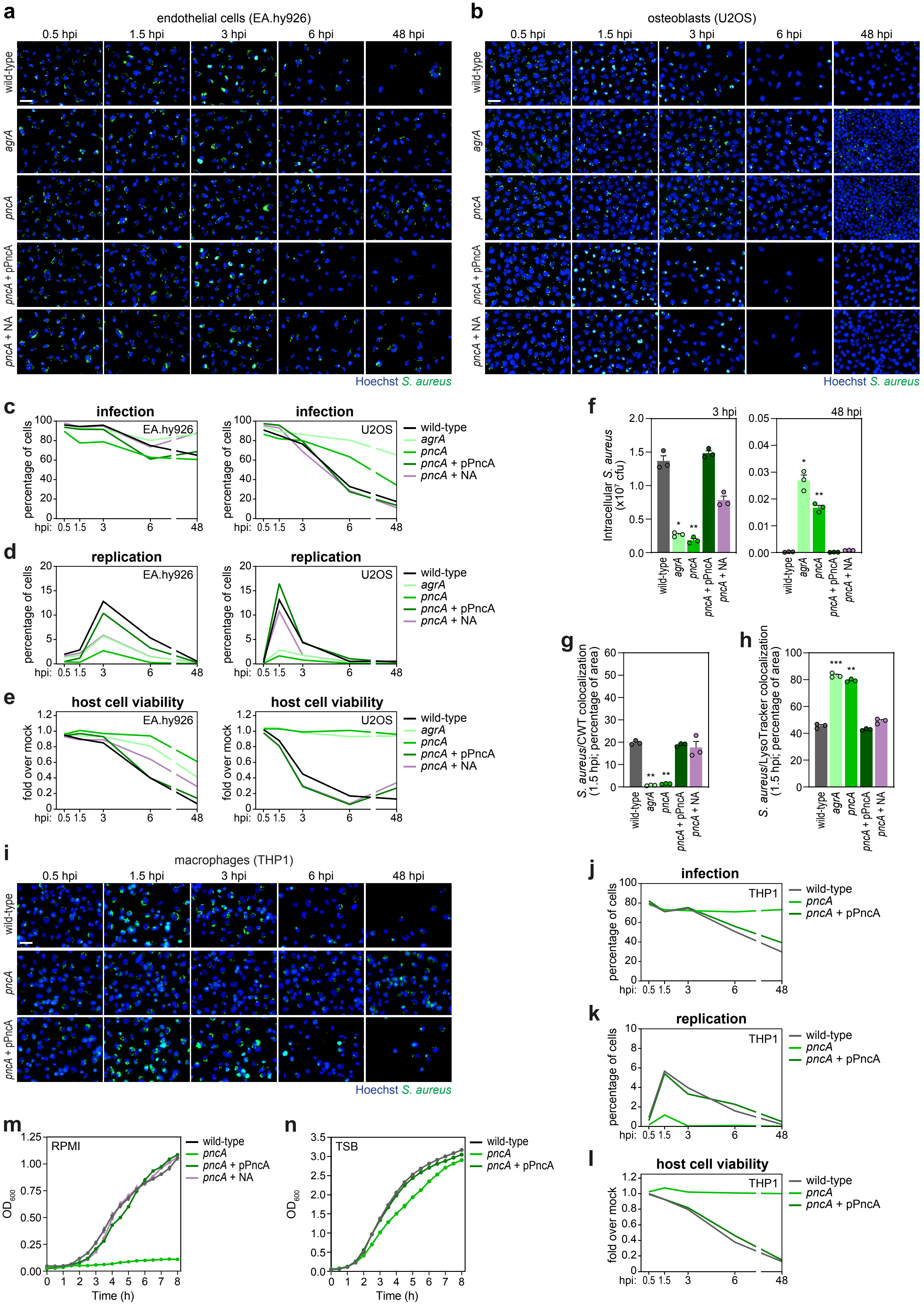

Supplementary Figure 9. *S. aureus pncA* mutant shows impaired intracellular replication, host cytotoxicity and vacuolar escape.
a-e. Representative fluorescence microscopy images (**a,b**) and time-course analysis of infection (**c**), intracellular replication (**d**), and host cell viability (**e**) upon infection of EA.hy926 and U2OS cells with *S. aureus* WT, mutant strains *agrA*, *pncA*, *pncA*+pPncA, and *pncA* in medium supplemented with nicotinic acid (*pncA*+NA). Results shown in panels c-e are the mean of 3 biologically independent experiments; microscopy images in panels a,b are representative of 3 biologically independent experiments. Scale bar, 50 µm.
f. Quantification of intracellular *S. aureus* by CFU assays upon infection of HeLa cells with *S. aureus* WT, *agrA*, *pncA*, *pncA*+pPncA, and *pncA*+NA, and analysed at 3 and 48 hpi. Results are presented as the mean ± s.e.m. of 3 biologically independent experiments; *P<0.05 and **P<0.01 (statistical analysis is detailed in Supplementary Data 4).
g and h. *S. aureus*/CWT (**g**) and *S. aureus*/LysoTracker (**h**) colocalization upon infection of HeLa cells with *S. aureus* WT, *agrA*, *pncA*, *pncA*+pPncA, and *pncA*+NA, analyzed at 1.5 hpi. Results are presented as the mean ± s.e.m. of 3 biologically independent experiments; **P<0.01 and ***P<0.001 (statistical analysis is detailed in Supplementary Data 4).
i-l. Representative fluorescence microscopy images (**i**) and time-course analysis of infection (**j**), intracellular replication (**k**), and host cell viability (**l**) upon infection of macrophages (differentiated THP1 cells) with *S. aureus* WT, mutant strains *pncA* and *pncA*+pPncA. Results shown in panels j-l are the mean of 3 biologically independent experiments; microscopy images are representative of 3 biologically independent experiments. Scale bar, 50 µm.
m and n. Growth curves of the *S. aureus* WT, *pncA*, *pncA*+pPncA, and *pncA*+NA in RPMI medium (**m**) and WT, *pncA*, *pncA*+pPncA in TSB medium (**n**). OD_{600} was measured at 30 min intervals for 8 h. For each strain, results are presented as the mean ± s.e.m. of 5 biologically independent experiments.
Source data are provided as a Source Data file.

Rodrigues Lopes *et al.* - Supplementary Figure 10

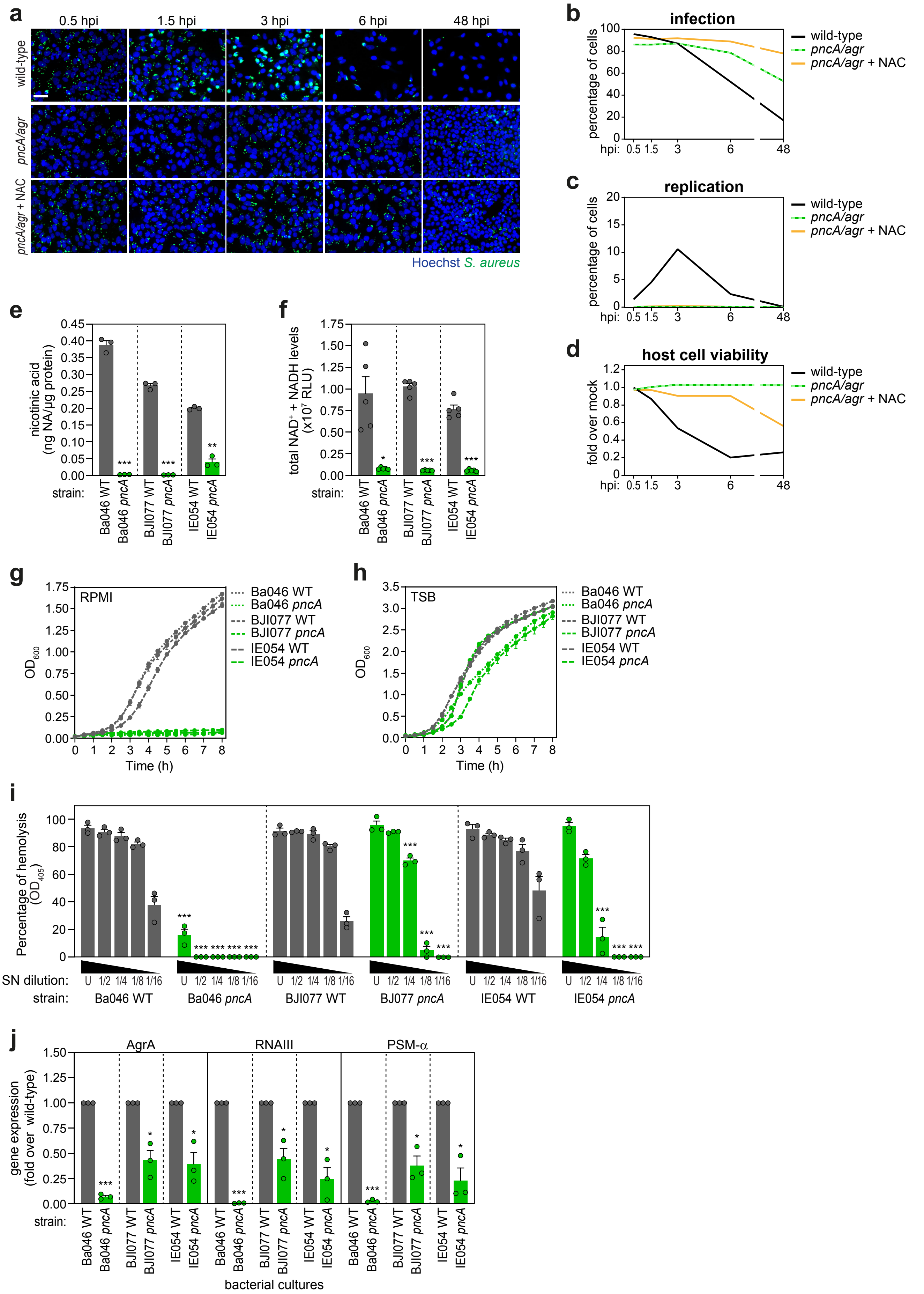

Supplementary Figure 10. *S. aureus pncA* mutants in clinical isolates show impaired *agr* activation.
a-d. Representative fluorescence microscopy images (**a**) and time-course analysis of infection (**b**), intracellular replication (**c**), and host cell viability (**d**) upon infection of HeLa cells with *S. aureus* WT, *pncA/agr* and *pncA/agr*+NAC. Results shown in panels b-d are the mean of 3 biologically independent experiments; microscopy images are representative of 3 biologically independent experiments. Scale bar, 50 μm.
e. Quantification by liquid chromatography-mass spectrometry of nicotinic acid in stationary phase cultures of *S. aureus* clinical isolates (Ba046, BJI077, and IE054) and corresponding *pncA* mutants. Results are presented as the mean ± s.e.m. of 3 biologically independent experiments; $**P<0.01$, and $***P<0.001$ (statistical analysis is detailed in Supplementary Data 4).
f. Quantification of total NAD^+ & NADH levels in stationary phase cultures of *S. aureus* clinical isolates (Ba046, BJI077, and IE054) and corresponding *pncA* mutants. Results are presented as the mean ± s.e.m. of 5 biologically independent experiments; $*P<0.05$, and $***P<0.001$ (statistical analysis is detailed in Supplementary Data 4).
g and h. Growth curves of the *S. aureus* clinical isolates (Ba046, BJI077, and IE054) and corresponding *pncA* mutants in RPMI (**g**) and TSB media (**h**). OD_{600} was measured at 30 min intervals for 8 h. For each strain, results are presented as the mean ± s.e.m. of 5 biologically independent experiments.
i. Hemolytic activity of *S. aureus* clinical isolates (Ba046, BJI077, and IE054) and corresponding *pncA* mutants by quantification of OD_{405} of a 2% sheep blood solution upon incubation with the strains supernatants. Culture supernatants were used undiluted (U) or after 2-fold serial dilutions (up to 1/16). All values are shown normalized to the OD_{405} of blood incubated with TSB containing 1% Triton X-100, and presented as mean ± s.e.m. of 3 biologically independent experiments; $***P<0.001$ (statistical analysis is detailed in Supplementary Data 4).
j. Expression levels of AgrA, RNAIII, and PSM-α determined by qRT-PCR in liquid cultures of *S. aureus* clinical isolates (Ba046, BJI077, and IE054) and corresponding *pncA* mutants. Results are shown normalized to *S. aureus* WT, and presented as mean ± s.e.m. of 3 biologically independent experiments; $*P<0.05$, and $***P<0.001$ (statistical analysis is detailed in Supplementary Data 4).
Source data are provided as a Source Data file.
