# Supplementary material for: Systematic identification of bacterial factors driving Staphylococcus aureus intracellular lifestyle in non-professional phagocytes
Source: Nat Commun. 2025 Dec 10;16:10907. doi: 10.1038/s41467-025-66373-9 (PMC12696020; doi:10.1038/s41467-025-66373-9)
Supplement: Supplementary file 2 — Description of Additional Supplementary Files [file 41467_2025_66373_MOESM2_ESM.pdf]

Description of Additional Supplementary Files

Supplementary Data 1. Complete results of the NTML screening to identify *S. aureus* factors relevant for the intracellular lifestyle within epithelial cells.
Results for the time course (0.5, 1.5, 3, 6, and 48 hpi) analysis of infection (percentage), intracellular replication (percentage), and host cell viability (normalized to mock-treated cells) for the 1,920 *S. aureus* mutants (Nebraska transposon mutant library) and WT strains in epithelial cells (HeLa cells). Results are shown for the 2 biologically independent experiments, including information about the OD_{600} of the bacterial growths used for infection, means for the various phenotypes, maximum mean values of *S. aureus* intracellular replication (percentage and normalized to *S. aureus* WT), minimum mean values of host cell viability (normalized to mock and normalized to *S. aureus* WT), infection at 48 hpi normalized to *S. aureus* WT, and viability at 3 hpi normalized to *S. aureus* WT.

Supplementary Data 2. Complete results for the phenotypic profiling of the 73 selected *S. aureus* mutant strains in four non-professional phagocytic cells.
Results for the time course (0.5, 1.5, 3, 6, and 48 hpi) analysis of infection (percentage), intracellular replication (percentage), and host cell viability (normalized to mock-treated cells) for *S. aureus* WT and the 73 mutants strains in 4 host cell types, specifically epithelial cells (HeLa), endothelial cells (EA.hy926), osteoblasts (U2OS), and primary endothelial cells (HUVEC). Results are shown for the 3 biologically independent experiments, including information about means for the various phenotypes, maximum mean values of *S. aureus* intracellular replication (percentage and normalized to *S. aureus* WT), minimum mean values of host cell viability (normalized to mock and normalized to *S. aureus* WT). Information about the phenotypic cluster of each mutant strain is shown.

Supplementary Data 3. Results for the cytosolic/vacuolar localization of the 73 selected *S. aureus* strains.
Results for percentage of *S. aureus*/CWT colocalization and of *S. aureus*/LysoTracker colocalization for epithelial cells (HeLa; 1.5 hpi), endothelial cells (EA.hy926; 1.5 hpi), and osteoblasts (U2OS; 0.5 hpi) infected with *S. aureus* WT and 73 selected mutant strains. Results are shown for the 3 biologically independent experiments and corresponding means.

Supplementary Data 4. Statistical analyses.
Detailed information about statistical analysis and p-values for group comparison
